# Iron-Doping of Copper Oxide Nanoparticles Lowers Their Toxic Potential on C6 Glioma Cells

**DOI:** 10.1007/s11064-020-02954-y

**Published:** 2020-01-29

**Authors:** Arundhati Joshi, Hendrik Naatz, Kathrin Faber, Suman Pokhrel, Ralf Dringen

**Affiliations:** 1 Center for Biomolecular Interactions Bremen, Faculty 2 (Biology/Chemistry), University of Bremen, PO. Box 330440, 28334 Bremen, Germany; 2 Center for Environmental Research and Sustainable Technology, Leobener Strasse 5, 28359 Bremen, Germany; 3 Faculty of Production Engineering, University of Bremen, Badgasteiner Str. 1, 28359 Bremen, Germany; 4 Leibniz Institute for Materials Engineering IWT, Badgasteiner Str. 3, 28359 Bremen, Germany

## Abstract

Copper oxide nanoparticles (CuO-NPs) are well known for their cytotoxicity which in part has been attributed to the release of copper ions from CuO-NPs. As iron-doping has been reported to reduce the susceptibility of CuO-NPs to dissolution, we have compared pure CuO-NPs and CuO-NPs that had been doped with 10% iron (CuO-Fe-NPs) for copper release and for their toxic potential on C6 glioma cells. Physicochemical characterization revealed that dimercaptosuccinate (DMSA)-coated CuO-NPs and CuO-Fe-NPs did not differ in their size or zeta potential. However, the redox activity and liberation of copper ions from CuO-Fe-NPs was substantially slower compared to that from CuO-NPs, as demonstrated by cyclic voltammetry and by the photometric quantification of the copper ion-bathocuproine complex, respectively. Exposure of C6 cells to these NPs caused an almost identical cellular copper accumulation and each of the two types of NPs induced ROS production and cell toxicity. However, the time- and concentration-dependent loss in cell viability was more severe for cells that had been treated with CuO-NPs compared to cells exposed to CuO-Fe-NPs. Copper accumulation and toxicity after exposure to either CuO-NPs or CuO-Fe-NPs was prevented in the presence of copper chelators, while neutralization of the lysosomal pH by bafilomycin A1 prevented toxicity without affecting cellular copper accumulation or ROS production. These data demonstrate that iron-doping does not affect cellular accumulation of CuO-NPs and suggests that the intracellular liberation of copper ions from CuO-NPs is slowed by the iron doping, which in turn lowers the cell toxic potential of iron-doped CuO-NPs.

## Introduction

Copper oxide nanoparticles (CuO-NPs) have gained a lot of attention for industrial applications due to their beneficial optical, electrical and thermal properties [1–4]. They are frequently applied as catalysts or additives [5–8] as well as for medical purposes due to their high antimicrobial potential [9]. Copper-containing NPs are generated and released not only into the outdoor environment during welding [10] or from anti-fouling paints [11], but also in indoor environments from household electric appliances like vacuum cleaners [12]. The toxicity of CuO-NPs has been extensively studied in vitro, in rat and human cell culture models [13, 14]. In addition, recent in vivo studies have focused on the toxicity of CuO-NPs internalized via oral or intranasal routes in test animals [15, 16] and on the potential risk of air-borne CuO-NPs to human health [17–19].

CuO-NP toxicity has been directly linked to intracellular release of copper ions from internalized CuO-NPs in cell culture studies [20–22]. However, CuO-NPs are rapidly dissolving [23, 24] and they release ionic copper even in the absence of cells [25–27]. Therefore, the copper ions released from dispersed CuO-NPs have been considered to strongly contribute to the copper toxicity [28, 29] and to the antibacterial effects [24] described for CuO-NPs. However, at least for glioma cells evidence has also been provided for a direct contribution of nanoparticulate copper in the cell toxicity observed after application of CuO-NPs [30].

The fast dissolution of CuO-NPs and the role of dissolved copper ions in cytotoxicity is a major concern in order to evaluate exclusive NP-mediated toxicity [23, 31]. The release of copper ions from CuO-NPs can be minimized or decelerated by modification of the procedures to synthesize the NPs [32], for example by doping of the CuO-NPs by other metals such as iron [33, 34]. Although iron-doping has been reported to decrease the colloidal stability of CuO-NPs in natural and synthetic waters [35], it has been demonstrated to improve the structural and thermodynamic stability of CuO-NPs and zinc oxide NPs [34, 36, 37]. Accordingly, iron-doping of CuO-NPs reportedly decreased their cytotoxicity in some peripheral cell lines, zebra fish embryos and sea urchin embryos [34, 38, 39]. To our knowledge, iron-doped CuO-NPs have not been investigated so far regarding their uptake and toxic potential on neural cells.

The C6 glioma cell line [40] has been frequently used to study the uptake and toxicity of metal-containing NPs [41–44], including CuO-NPs [29, 30, 45]. In order to compare uptake and cell toxic potential of CuO-NPs and iron-doped CuO-NPs, we generated iron-free CuO-NPs as well as CuO-NPs that contained 10 molar % iron (CuO-Fe-NPs), coated these NPs with dimercaptosuccinate (DMSA), investigated their physicochemical properties and stability and compared their cytotoxic potential on C6 glioma cells. Our data confirm that iron-doping indeed slowed copper ion release from CuO-NPs for the conditions applied and revealed for C6 cells a lower cell toxic potential of the iron-doped CuO-NPs compared to the iron-free NPs, although the copper accumulation was not affected by the absence or the presence of iron in the NPs.

## Materials

Fetal calf serum (FCS), trypsin solution and penicillin/streptomycin solution were obtained from Biochrom (Berlin, Germany) and Dulbecco’s modified Eagle’s medium (DMEM) from Gibco (Karlsruhe, Germany). Copper chloride, potassium chloride, magnesium chloride hexahydrate and sodium bicarbonate were purchased from Riedel-de Haën (Seelze, Germany). Copper naphthenate, iron naphthenate, xylene and ethanol (AR grade) were purchased from Strem Chemicals (Newburyport, USA). 4-(2-Hydroxyethyl)-1-piperazine ethanesufonic acid (HEPES) was obtained from Roth (Karlsruhe, Germany) and bafilomycin A1 was obtained from Enzo Life Sciences (Lörrach, Germany). Bovine serum albumin (BSA), NADH and sodium ascorbate were purchased from Applichem (Darmstadt, Germany). Triton X-100, sodium hydroxide, dihydrorhodamine 123 and potassium phosphate were purchased from Fluka (Buchs, Switzerland). Folin–Ciocalteau’s reagent, calcium chloride dihydrate, d-glucose, disodium hydrogen phosphate, sodium pyruvate, nitric acid, dipotassium phosphate trihydrate, 65% HNO_3_ (suprapur), 35% H_2_O_2_, copper standard solution, iron standard solution as well as palladium-matrixmodifier were purchased from Merck (Darmstadt, Germany). Sodium chloride, 2,3-dimercaptosuccinic acid (DMSA), bathocuproine disulfonic acid disodium salt (BCS), ammonium tetrathiomolybdate (TTM), bisbenzimide Hoechst 33342 (H33342) and fluorine doped tin oxide (FTO) glass (sheet resistance 7 Ω/sq) were purchased from Sigma-Aldrich (Steinheim, Germany). 24-Well cell culture plates and 96-well microtiter plates were obtained from Sarstedt (Nümbrecht, Germany).

## Methods

### Synthesis and DMSA-Coating of CuO-NPs and CuO-Fe-NPs

CuO-NPs and 10% iron-doped CuO-NP (CuO-Fe-NPs) ultrafine powders were produced via flame spray pyrolysis as described earlier [34]. Briefly, copper naphthenate and iron naphthenate precursor solutions (0.5 M in xylene) were fed to the flame spray pyrolysis nozzle at 5 mL/min using a syringe pump (KD Scientific, KDS 100) and dispersed using 5 L/min of oxygen with a pressure drop of 0.15 MPa at the nozzle exit. A methane (1.5 mL/min)/oxygen (3.2 mL/min) premixed support flame was used for ignition and combustion. After formation in the combustion process, the nanoparticles were collected on a glass fiber filter (Pall, Type A/E) which was placed 60 cm above the nozzle. Gas flow rates were adjusted with calibrated mass flow controllers (Bronkhorst, E-7500-RBB, Kamen, Germany).

For DMSA coating, 20 mg of the ultrafine CuO-NP- or CuO-Fe-NP-powder was dispersed in 10 mL water by sonication for 30 s at 50 W with a Branson B-12 sonifier (Danbury, Connecticut, USA). Subsequently, after addition of 10 mL of a pre-warmed (60 °C) 10 mM DMSA solution, the dispersion was sonicated again for 5 min at 50 W. The resulting DMSA-coated NP dispersion was then centrifuged with a high salt buffer (20 mM HEPES/NaOH buffer pH 7.4 containing 1.45 M NaCl, 18 mM CaCl_2_, 54 mM KCl, and 10 mM MgCl_2_) for 10 min at 1500* g* to obtain a NP pellet. The supernatant containing free DMSA molecules was discarded and the pellet containing DMSA-coated NPs was washed with water, followed by a subsequent centrifugation step to remove the remaining washing solution. The resulting DMSA-coated NPs were dispersed in H_2_O, sonicated for 30 s at 50 W and stored at 4 °C.

The total copper and iron contents of the NP dispersions were determined after dissolution of the NPs with concentrated HNO_3_ by graphite furnace atomic absorption spectroscopy (AAS) using a Varian (Darmstadt, Germany) AA-240Z spectrophotometer and a GTA-120 graphite-tube atomizer equipped with a PSD-120 programmable sample dispenser (Software Varian SpectrAA 5.01). The optimized instrumental conditions for the quantification of copper were as previously described [46, 47]: wavelength: 327.4 nm, slit width: 0.5 nm lamp current: 7 mA, activated Zeeman background correction, protective gas: argon, absorbance measurement: peak area, using a copper standard (1 g/L Cu(NO_3_)_2_ in HNO_3_) in a calibration range of 0—1.5 µM. The optimized instrumental conditions for the quantification of iron were: wavelength: 248.3 nm, slit width: 0.2 nm, lamp current: 5 mA, activated Zeeman background correction, protective gas: argon, absorbance measurement: peak area, using an iron standard (1 g/L Fe(NO_3_)_3_ in HNO_3_) in a calibration range of 0—0.9 µM.

The absence of iron in the CuO-NP dispersions was confirmed by measurement of total iron in the dispersed NPs. Iron represented less than 0.002% of the total copper content of the CuO-NP dispersions, while CuO-Fe-NP dispersions contained iron in a molar ratio of 12 ± 2% (*n* = 3) compared to the total copper content of the respective dispersions. Concentrations of NPs applied to cells refer to the concentration of total copper of the NP dispersions and not to the concentration of particles.

### Characterization of the CuO-NPs and CuO-Fe-NPs

For transmission electron microscopy (TEM) analysis, uncoated NPs were deposited onto carbon-coated silicon nitride grids (SimPore, West Hendrietta, New York, USA) using one droplet of the dispersion (NPs suspended in ethanol), followed by drying at room temperature. Subsequently, TEM analysis was carried out on a FEI Titan 80-300 ST microscope (FEI company, Hillsboro, Oregon, USA) using an acceleration voltage of 300 kV.

Physicochemical properties like the hydrodynamic diameter and zeta potential of DMSA-coated NP dispersions in water and physiological medium (IB-BSA; 20 mM HEPES, 145 mM NaCl, 5 mM d-glucose, 1.8 mM CaCl_2_, 5.4 mM KCl, 1 mM MgCl_2_, 0.5 mg/mL BSA; pH 7.4) were determined by dynamic (DLS) and electrophoretic light scattering (ELS) in a Beckman Coulter (Krefeld, Germany) Delsa Nano C Particle Analyzer at 25 °C, as earlier described [46].

### Assessment of Liberation of Ionic Copper from CuO-NPs or CuO-Fe-NPs

Ionic copper liberated from DMSA-coated CuO-NPs and CuO-Fe-NPs after application of an excess of the copper chelator bathocuproine disulfonate (BCS) [48] was investigated by determining the absorbance of the copper-BCS complex formed at 484 nm as previously described [29, 49]. Briefly, 100 µM CuO-NPs or CuO-Fe-NPs (or dissolved CuCl_2_ as control for ionic copper) in IB-BSA were mixed with IB-BSA containing 1 mM BCS, in the absence or presence of 1 mM of the reducing agent ascorbate at room temperature in wells of a 96-well microtiter plate. Subsequently, the absorbance of the generated copper-BCS complex was monitored for up to 30 min at 484 nm and an end-point absorbance was recorded after 24 h in a MultiSkan Sky microtiter plate photometer (Life Technologies, Darmstadt, Germany).

### Electrochemical Characterization of CuO-NPs and CuO-Fe-NPs via Cyclic Voltammetry

Electrochemical characterization of DMSA-coated CuO-NPs and CuO-Fe-NPs was carried out in a three-electrode setup using an Ag/AgCl reference electrode (SI Analytics, Mainz, Germany) and a Pt-coated grid as counter electrode. For preparation of the working electrodes, the NPs were collected on a glass fiber filter (Pall, Type A/E) during flame spray pyrolysis and transferred to electrically conductive FTO glass (7 Ω/sq) using a lamination process with a compaction pressure of 3.2 MPa, as described earlier [50]. Subsequently, NPs were stabilized on the electrodes at 350 °C for 4 h. Cyclic voltammetry was recorded at a scan rate of 50 mV s^−1^ between −0.6 and 0.8 V versus Ag/AgCl using a potentiostat (VMP300, Biologic and Software EC-Lab, Seyssinet-Pariset, France) as previously described [34].

### Cell Culture and Experimental Incubations

The C6 glioma cell line was kindly provided by Dr. Frank Dietz (University of Bremen). These cells express the astrocytic marker protein glial-fibrillary acidic protein [51] and were cultured as described earlier [29]. From a 175 cm^2^ cell-culture flask, 80% confluent cultures were harvested and were seeded in 1 mL culture medium (90% DMEM containing 25 mM d-glucose, 1 mM sodium pyruvate, 18 U/mL penicillin G, 18 µg/mL streptomycin sulphate and 10% FCS; pH 7.4) at a density of 200,000 viable cells per mL into wells of 24-well culture plates. After 24 h, cells were washed twice with 1 mL of pre-warmed (37 °C) incubation buffer (IB-BSA). Subsequently, cells were incubated for the indicated time periods at 37 °C in the humidified atmosphere of an incubator (without CO_2_ supply) with 200 µL IB-BSA containing DMSA-coated CuO-NPs or CuO-Fe-NPs in the concentrations given in the legends of the figures and tables. Following the incubations, the cells were washed twice with 1 mL pre-warmed (37 °C) IB-BSA to subsequently determine cellular LDH activity as an indicator for cell viability. Alternatively, for quantification of cellular metal and protein contents, cells were washed with 1 mL ice cold (4 °C) phosphate buffered saline (PBS; 10 mM potassium phosphate buffer containing 150 mM sodium chloride, pH 7.4) and were stored dry at − 20 °C till the measurements were done.

### Quantification of Cellular Copper and Iron Contents

Cells were lysed in 400 µL 50 mM NaOH in water for 2 h in the wells of 24-well plates and 100 µL of the cell lysate were ashed and used for measurements of cellular iron and cellular copper by AAS using the settings described above. For the measurement of cellular iron, 100 µL cell lysate was mixed with 100 µL of 65% HNO_3_ whereas for the measurement of cellular copper, 100 µL cell lysate was mixed with 100 µL of a 1:1 mixture of 35% H_2_O_2_ and 65% HNO_3_. These mixtures were incubated at 65 °C for 60 min and subsequently heated at 85 °C overnight to ash the organic part of the samples. The dry residues were dissolved in 100 µL of 0.1% (v/v) HNO_3_ for iron and copper quantification via AAS. The total volume injected (17 µL) contained 10 µL of sample, 2 µL matrix modifier (10.0 ± 0.2 g/L Pd(NO_3_)_2_ in HNO_3_) and 5 µL 0.1% (v/v) HNO_3_. The cellular copper and iron contents were normalized to the protein content of each respective sample to obtain the specific cellular copper or iron contents expressed as nmol/mg protein.

### Tests for Cell Viability and Protein Content

The activity of the cellular lactate dehydrogenase (LDH) was determined as an indicator of cell viability as described previously [52]. Cells were lysed after a given treatment with 1% (v/v) Triton X-100 in 200 µL IB-BSA for 30 min and a 10 µL aliquot of this lysate was used in wells of a 96-well plate for measurement of the cellular LDH activity.

The protein content of the cultures was quantified according to the Lowry method [53], using BSA as a standard. 100 µL of the cell lysate, which was obtained after lysing the cells in 400 µL 50 mM NaOH for 2 h in the wells of a 24-well plate, was used to determine protein content.

### Staining of Cell Cultures for Cellular Reactive Oxygen Species (ROS)

Cells were stained with dihydrorhodamine 123 to investigate the presence of an increased amount of cellular reactive oxygen species (ROS) as described previously [54]. After a given incubation, cells were washed twice with pre-warmed (37 °C) IB and subsequently incubated for 30 min at 37 °C in 200 µL IB (without glucose) containing the fluorescent dyes dihydrorhodamine 123 (5 µg/mL) and Hoechst H33342 (10 µM). Subsequently, the cells were washed twice with pre-warmed (37 °C) IB and analyzed directly for their fluorescence on the Eclipse TE2000-U fluorescent microscope using the appropriate filter settings: rhodamine123 (λ_ex_: 465–495 nm; λ_em_: 505–515 nm; dichromatic mirror: 505 nm) and H33342 (λ_ex_: 330–380 nm; λ_em_: 435–485 nm; dichromatic mirror: 400 nm).

### Presentation of Data

Quantitative data shown in the figures and tables represent means ± standard deviations (SD) of values derived from *n* independent experiments. Cell experiments were performed in triplicates in *n* independent experiments using different cell passages. Cell images showing cytochemical staining are from a representative experiment that was reproduced once in an independent experiment with a similar outcome. Statistical analysis between multiple groups of data was performed by ANOVA followed by the Bonferroni’s post hoc test, whereas statistical analysis between two sets of data was performed by the paired Student’s *t *test using the program GraphPad InStat. Values of p > 0.05 were considered not significant.

## Results

### Physicochemical Characterization of CuO-NPs and CuO-Fe-NPs

**Fig. 1:**
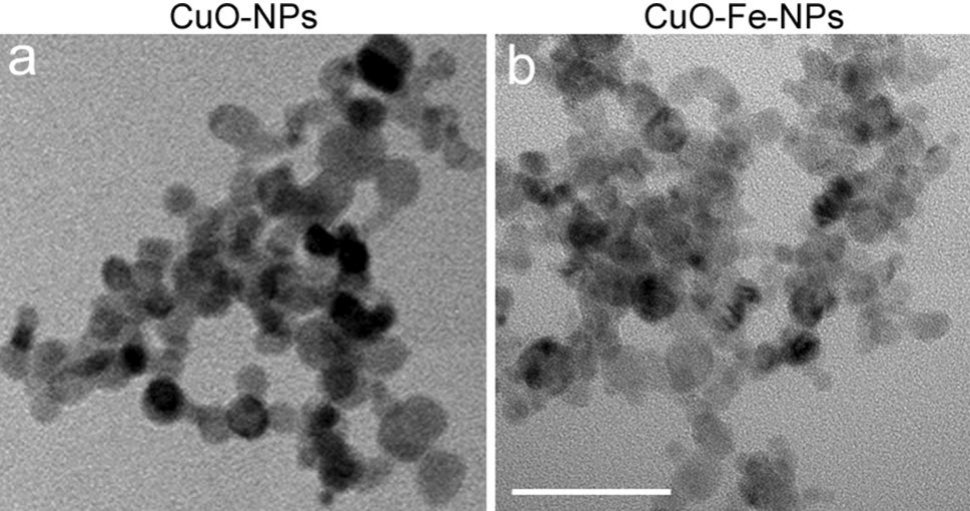
Visualization of NPs. The crystalline structure of uncoated CuO-NPs (**a**) and CuO-Fe-NPs (**b**) was visualized by transmission electron microscopy. The scale bar in b represents 50 nm and applies to both panels

CuO-NPs and CuO-Fe-NPs were synthesized by flame spray pyrolysis [34]. TEM analyses of the synthesized CuO-NPs (Fig. 1a) and CuO-Fe-NPs (Fig. 1b) showed an aggregated structure of highly crystalline primary particles of a diameter of around 10 nm, which is consistent with literature data for such NPs [34]. For application to cells, the synthesized NPs were coated with DMSA and the physicochemical properties of the NP dispersions in water and in the physiological medium (IB-BSA) used for cell experiments were determined. Characterization of CuO-NPs and CuO-Fe-NPs with DLS and ELS in H_2_O revealed a hydrodynamic NP diameter of around 209 nm and 210 nm and zeta (ζ) potentials of − 40.3 mV and − 37.2 mV, respectively (Table 1). When these NPs had been dispersed in IB-BSA, the hydrodynamic diameters of CuO-NPs and CuO-Fe-NPs were found significantly increased to 235 nm and 237 nm, respectively (Table 1), while the ζ-potential of both CuO-NPs and CuO-Fe-NPs became with − 11.8 mV and − 12.7 mV, respectively, more positive compared to the values determined for NP dispersions in H_2_O (Table 1). For the dispersions investigated, the dispersed CuO-NPs and CuO-Fe-NPs did not differ in their physicochemical properties. The polydispersity index of CuO-NPs and CuO-Fe-NPs dispersed in either H_2_O or IB-BSA was always below a value of 0.2, indicating a narrow aggregate size distribution (Table 1).

**Table 1:** Physicochemical characterization of DMSA-coated CuO-NPs and CuO-Fe-NPs

	CuO-NPs		CuO-Fe-NPs		*n*
	H_2_O	IB-BSA	H_2_O	IB-BSA	
Hydrodynamic diameter (nm)	209 ± 30	235 ± 35^*###*^	210 ± 31	247 ± 57^*##*^	7
Polydispersity index	0.14 ± 0.02	0.16 ± 0.03	0.18 ± 0.04	0.18 ± 0.04	7
ζ potential (mV)	− 40.3 ± 2.3	− 11.8 ± 3.3^*##*^	− 37.2 ± 2.6	− 12.7 ± 1.6^*##*^	3

CuO-NPs and CuO-Fe-NPs were coated with DMSA and subsequently the hydrodynamic diameter, polydispersity index and zeta potential were determined for dispersions (1 mM) in H_2_O or in IB-BSA. The data shown were obtained for NPs from *n* independently prepared dispersions. Hashes indicate the significance of differences of data obtained for dispersions in H_2_O and IB-BSA (^**##**^p < 0.01, ^**###**^p < 0.001; paired *t *test)

### Test for the Stability and Copper Ion Liberation from CuO-NPs or CuO-Fe-NPs

**Fig. 2:**
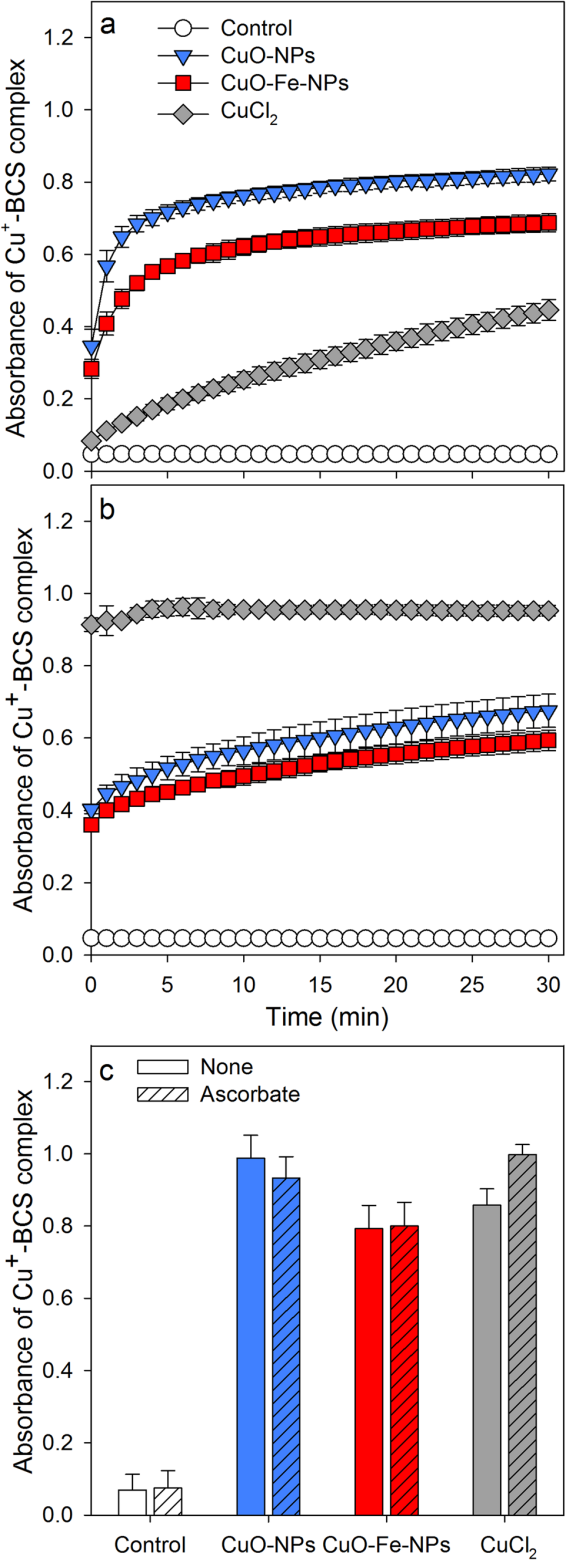
Liberation of copper ions from CuO-NPs and CuO-Fe-NPs in the presence of the copper chelator BCS. CuO-NPs, CuO-Fe-NPs or CuCl_2_ in concentrations of 100 µM in IB-BSA were mixed with 1 mM BCS (in IB-BSA) in the absence (**a**) or the presence of 1 mM ascorbate (**b**) and the increase in absorbance of the Cu^+^-BCS complex at a wavelength of 484 nm was monitored for up to 30 min (**a**, **b**) or recorded after 24 h of incubation (**c**). The data shown represent means ± SD of values obtained in three independent experiments

Iron-doped CuO-NPs have been reported to be less susceptible to dissolution in dispersion than iron-free CuO-NPs [34]. To test for the liberation of copper ions from NPs dispersed in the physiological medium used in the present study for all experiments (IB-BSA), the formation of the copper-BCS complex was photometrically monitored. Application of an excess of BCS to CuO-NPs caused an initial rapid increase in absorbance (within 1 min) at 484 nm to values of around 0.6, while for CuO-Fe-NPs the respective initial increase reached only an absorbance of around 0.4 (Fig. 2a). After the initial first minute of reaction, a similar gradual and slow time-dependent increase in the absorbance of the copper-BCS complex was observed for both types of NPs investigated reaching absorbances between 0.6 and 0.8 after 30 min (Fig. 2a), and absorbances between 0.8 and 1 after 24 h (Fig. 2c). The formation of the copper-BCS complex in NP dispersions was hardly affected by the absence or the presence of ascorbate (Fig. 2a–c), while the presence of ascorbate (Fig. 2b) facilitated immediate formation of the copper-BCS complex in the presence of ionic copper (Fig. 2b), compared to a slow formation of the complex in the absence of ascorbate (Fig. 2a). Almost identical end-point absorbances of the copper-BCS complexes were recorded after 24 h incubation of CuO-NPs, CuO-Fe-NPs or CuCl_2_ with BCS irrespective of the presence of ascorbate (Fig. 2c).

**Fig. 3:**
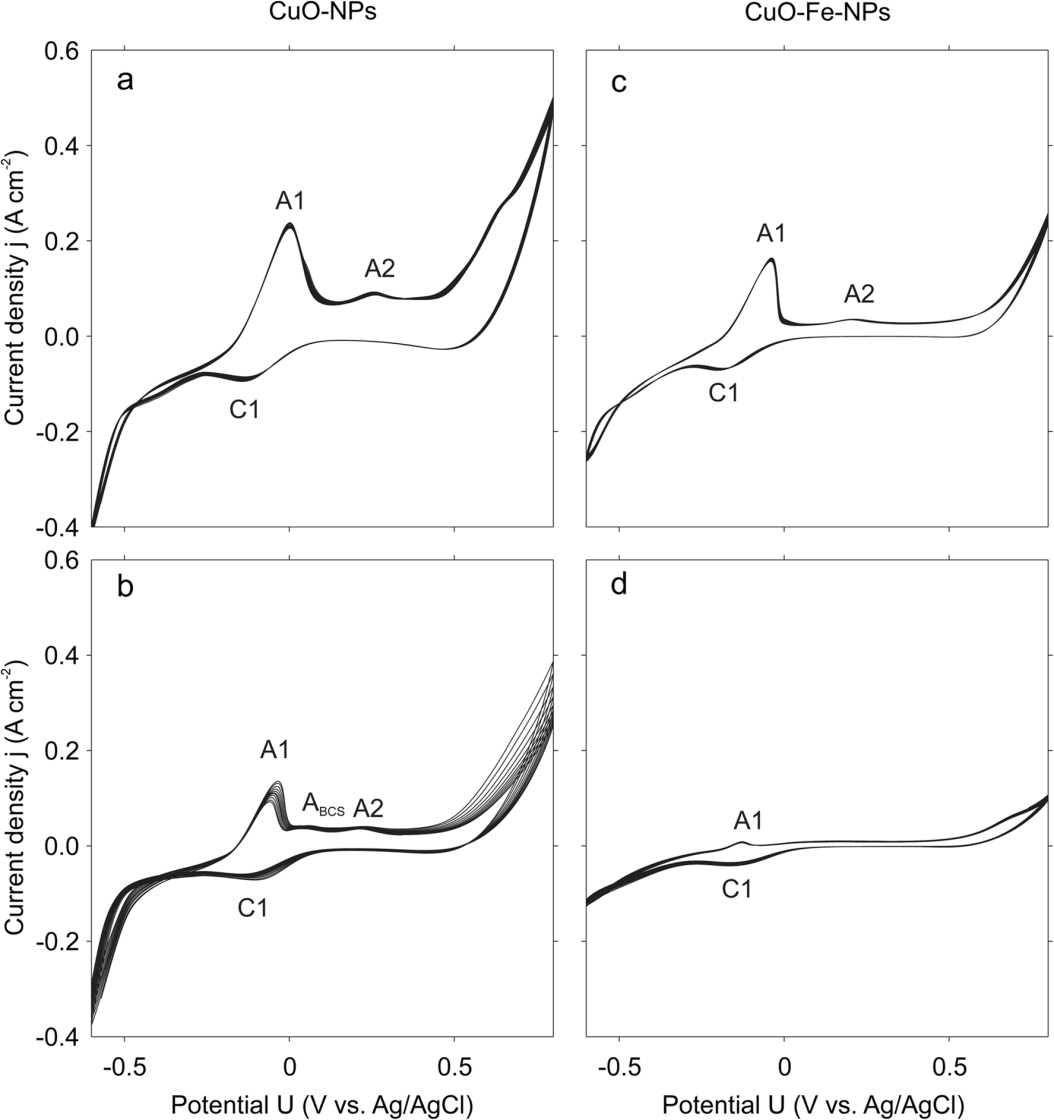
Electrochemical characterization of CuO-NPs and CuO-Fe-NPs by cyclic voltammetry. CuO-NPs (**a**, **b**) or CuO-Fe-NPs (**c**, **d**) were dispersed in IB-BSA in a concentration of 175 µM and deposited onto the electrodes. The cyclic voltammetry measurements were recorded in the absence (**a**, **c**) or the presence of 500 µM BCS (**b**, **d**) at 25 mV s^−1^. A1 and A2 denote anodic peaks and C1 denotes a cathodic peak observed at the electrodes. The data shown in the figure have been taken from a representative experiment

The characterization of CuO-NP and CuO-Fe-NP-containing electrodes by cyclic voltammetry in IB-BSA revealed substantial differences in the redox properties of CuO-NPs and CuO-Fe-NPs (Fig. 3). CuO-NPs showed a strong interaction with IB-BSA, with two distinguishable anodic peaks (Fig. 3a; A1, A2) and a cathodic peak (Fig. 3a; C1), indicating oxidation and reduction reactions respectively on the CuO-NP surface with components present in IB-BSA. These peaks were absent or less prominent for experiments in deionized water with 50 mM Na_2_SO_4_, which was added to achieve sufficient electrical conductivity (data not shown). Although similar anodic (A1, A2) and cathodic (C1) peaks were observed after the addition of the copper chelator BCS to IB-BSA, the interaction of CuO-NPs with components in IB-BSA was strongly reduced most likely due to interference by BCS (anodic peak A_BCS_) (Fig. 3b). CuO-Fe-NPs in IB-BSA as dispersant showed a very similar redox behavior as CuO-NPs as indicated by similar anodic and cathodic reactions, although the peak intensity was found reduced for CuO-Fe-NPs (Fig. 3c). This was especially observed for conditions containing BCS (Fig. 3d), which almost completely prevented the detection of anodic and cathodic peaks for CuO-Fe-NPs (Fig. 3d).

### Copper Accumulation and Toxicity After Application of CuO-NPs or CuO-Fe-NPs to C6 Glioma Cells

**Fig. 4:**
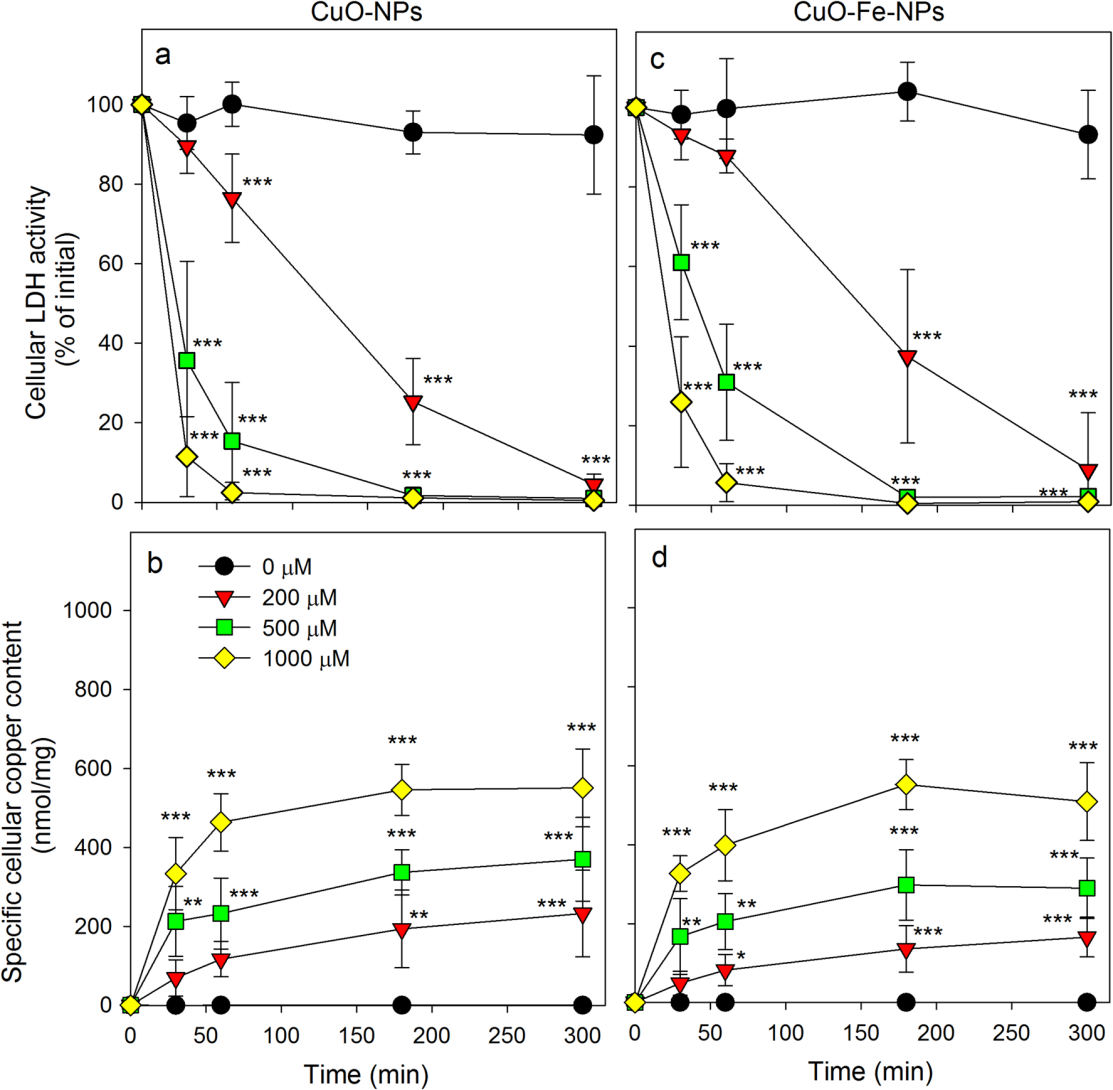
Consequences of an application of iron-free CuO-NPs and CuO-Fe-NPs on the viability and the copper content of C6 glioma cells. The cells were treated without or with CuO-NPs (**a**, **b**) or CuO-Fe-NPs (**c**, **d**) dispersed in the concentrations indicated in IB-BSA for up to 5 h at 37 °C before the cellular LDH activity (**a**, **c**) and the cellular copper content (**b**, **d**) were determined. The data shown represent means ± SD of values obtained in five independent experiments. Asterisks indicate the significance of differences of data compared with those of control cells (incubated in the absence of NPs) (*p < 0.05, **p < 0.01, ***p < 0.001; ANOVA)

To study the consequences of an application of CuO-NPs or CuO-Fe-NPs to C6 glioma cells, the cells were incubated in the absence or in the presence of up to 1000 µM CuO-NPs or CuO-Fe-NPs for up to 5 h (Fig. 4). In the absence of NPs, the cell viability was not compromised during an incubation for up to 5 h (Fig. 4a, c) and the initial specific cellular copper content (0.197 ± 0.20 nmol/mg; *n* = 5) was not altered. However, this basal copper content quickly increased after exposure of C6 cells to either CuO-NPs (Fig. 4b) or CuO-Fe-NPs (Fig. 4d) and the viability of the cells was compromised in a time- and concentration-dependent manner for both types of NPs applied, as indicated by the loss in cellular LDH activity (Fig. 4a, c). Already application of 200 µM CuO-NPs or CuO-Fe-NPs caused severe toxicity with maximal loss in LDH activity observed after 5 h of incubation (Fig. 4a, c), which was accompanied by a gradual increase in the specific cellular copper content to values of up to 200 nmol/mg (Fig. 4b, d). Application of higher concentrations of NPs (500 µM and 1000 µM) caused a more rapid increase in specific cellular copper contents (Fig. 4b, d) and a very rapid loss in cell viability (Fig. 4a, c).

**Fig. 5:**
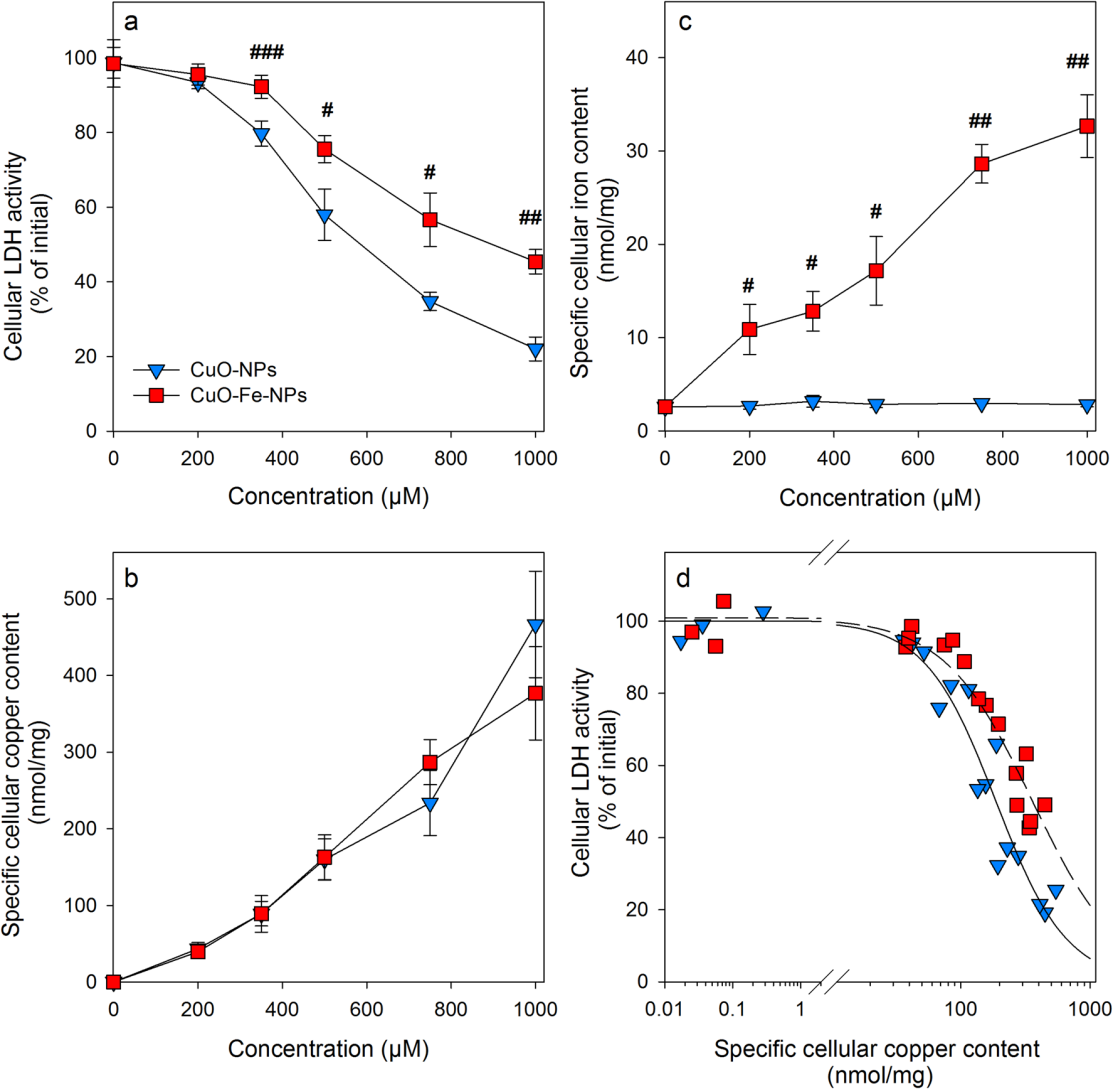
Comparison of copper accumulation and cell viability after application of iron-free CuO-NPs or CuO-Fe-NPs to C6 glioma cells. The cells were incubated with the indicated concentrations of CuO-NPs or CuO-Fe-NPs in IB-BSA for 30 min at 37 °C before the cellular LDH activity (**a**), the cellular copper content (**b**) and the cellular iron content (**c**) were determined. The data shown represent means ± SD of values obtained in three independent experiments. Hashes indicate the significance of differences of data obtained from cells that had been treated with CuO-NPs and CuO-Fe-NPs (^#^p < 0.05, ^##^p < 0.01, ^###^p < 0.001; paired *t *test). **d** Correlation of the individual values of cellular LDH activity from three independent experiments with the respective specific cellular copper contents determined for cells that had been treated with either CuO-NPs or CuO-Fe-NPs

In order to directly compare the acute consequences of an application of CuO-NPs or CuO-Fe-NPs on the viability and the cellular metal contents of C6 cells, the cells were incubated with CuO-NPs or CuO-Fe-NPs in concentrations of up to 1000 µM for 30 min (Fig. 5). The viability of C6 cells that had been exposed for 30 min to 200 µM CuO-NPs or CuO-Fe-NPs was not compromised (Fig. 5a) and the cells contained identical specific cellular copper contents of around 50 nmol/mg (Fig. 5b). In contrast, application of higher concentrations (400 µM to 1000 µM) of CuO-NPs or CuO-Fe-NPs caused a concentration-dependent decline in cell viability (Fig. 5a). However, the values of cellular LDH activity determined were for each concentration of NPs applied significantly lower by 20% to 40% for CuO-NP-treated cells compared to cells that had been exposed to CuO-Fe-NPs (Fig. 5a). For example cellular LDH activity was lowered to around 45% of the initial activity 30 min after application of 1000 µM CuO-Fe-NPs, while this concentration of CuO-NPs had lowered the detectable cellular LDH activity to 20% of the initial activity (Fig. 5a). A half-maximal loss in cellular LDH activity was observed 30 min after exposure of C6 cells to around 600 µM of CuO-NPs and to around 1000 µM of CuO-Fe-NPs (Fig. 5a).

In contrast, the specific cellular copper content increased almost proportional with the applied concentration of CuO-NPs and CuO-Fe-NPs reaching values of around 400 nmol/mg after incubation for 30 min with 1000 µM NPs (Fig. 5b). However, no significant differences were observed in the specific cellular copper contents of cells that had been treated with either of the two types of NPs (Fig. 5b). In addition, the specific cellular iron contents of CuO-NPs-treated C6 cells remained low and unchanged compared to values determined for control cells, while the specific cellular iron content of CuO-Fe-NP-treated cells was found increased in a concentration-dependent manner to values of around 30 nmol/mg found after incubation for 30 min with 1000 µM CuO-Fe-NPs (Fig. 5c).

Correlation of the decline in cellular LDH activity as indicator for a loss in cell viability of C6 cells that had been treated with CuO-NPs or CuO-Fe-NPs with the respective specific cellular copper contents (Fig. 5d) confirmed the higher toxic potential of CuO-NPs compared to CuO-Fe-NPs. Half-maximal impairment of C6 cell viability after application of CuO-NPs was found for cells that contained a specific cellular copper content of 223 ± 17 nmol/mg, while half-maximal toxicity for CuO-Fe-NPs treated cells was observed for a specific copper content of 332 ± 35 nmol/mg which represented a significant (*n* = 3, p < 0.05) increase by around 50%.

### Effects of Temperature on Copper Accumulation and Cell Toxicity of C6 Cells Exposed to CuO-NPs or CuO-Fe-NPs

To assess the effect of the incubation temperature on the cellular copper accumulation of CuO-NP- or CuO-Fe-NP-treated C6 glioma cells, the cells were incubated for 30 min at 37 °C or 4 °C with 1 mM CuO-NPs or CuO-Fe-NPs (Table 2). The viability of control cells that had been treated in the absence of NPs at 37 °C or 4 °C was not compromised and these cells contained very low amounts of copper (Table 2). As shown before (Fig. 4a, c, 5a), a 30 min incubation of C6 cells with 1 mM CuO-NPs or CuO-Fe-NPs at 37 °C severely compromised cell viability as demonstrated by a severe loss of cellular LDH activity (Table 2) that was accompanied with a strong copper accumulation to specific cellular copper contents of around 500 nmol/mg. In contrast, respective incubations at 4 °C prevented the NP-induced loss in cellular LDH activity almost completely and reduced the increase in specific cellular copper content compared to the values determined for 37 °C incubations to 20%, which most likely represents NPs attached extracellularly to the cell membrane [46, 55].

**Table 2:** Effects of temperature on the copper accumulation and the viability of NP-exposed C6 glioma cells

	Cellular LDH activity (% of initial)		Specific cellular copper content (nmol/mg protein)	
	37 °C	4 °C	37 °C	4 °C
Control	97 ± 1	90 ± 4	0.03 ± 0.01	0.08 ± 0.1
CuO-NPs	7 ± 4	86 ± 7^**##**^	532 ± 65	102 ± 23^**##**^
CuO-Fe-NPs	18 ± 9	90 ± 4^**##**^	457 ± 46	88 ± 6^**##**^

C6 cells were incubated without (control) or with 1 mM CuO-NPs or CuO-Fe-NPs in IB-BSA for 30 min at 37 °C or 4 °C before the cellular LDH activity and specific cellular copper contents were determined. The data shown represent means ± SD of values obtained in three independent experiments. Hashes indicate the significance of differences of data from cells that had been treated at 37 °C or at 4 °C (^**##**^p < 0.01; paired *t *test)

### Copper Chelators Prevent Copper Accumulation and Toxicity of CuO-NPs and CuO-Fe-NPs

**Fig. 6:**
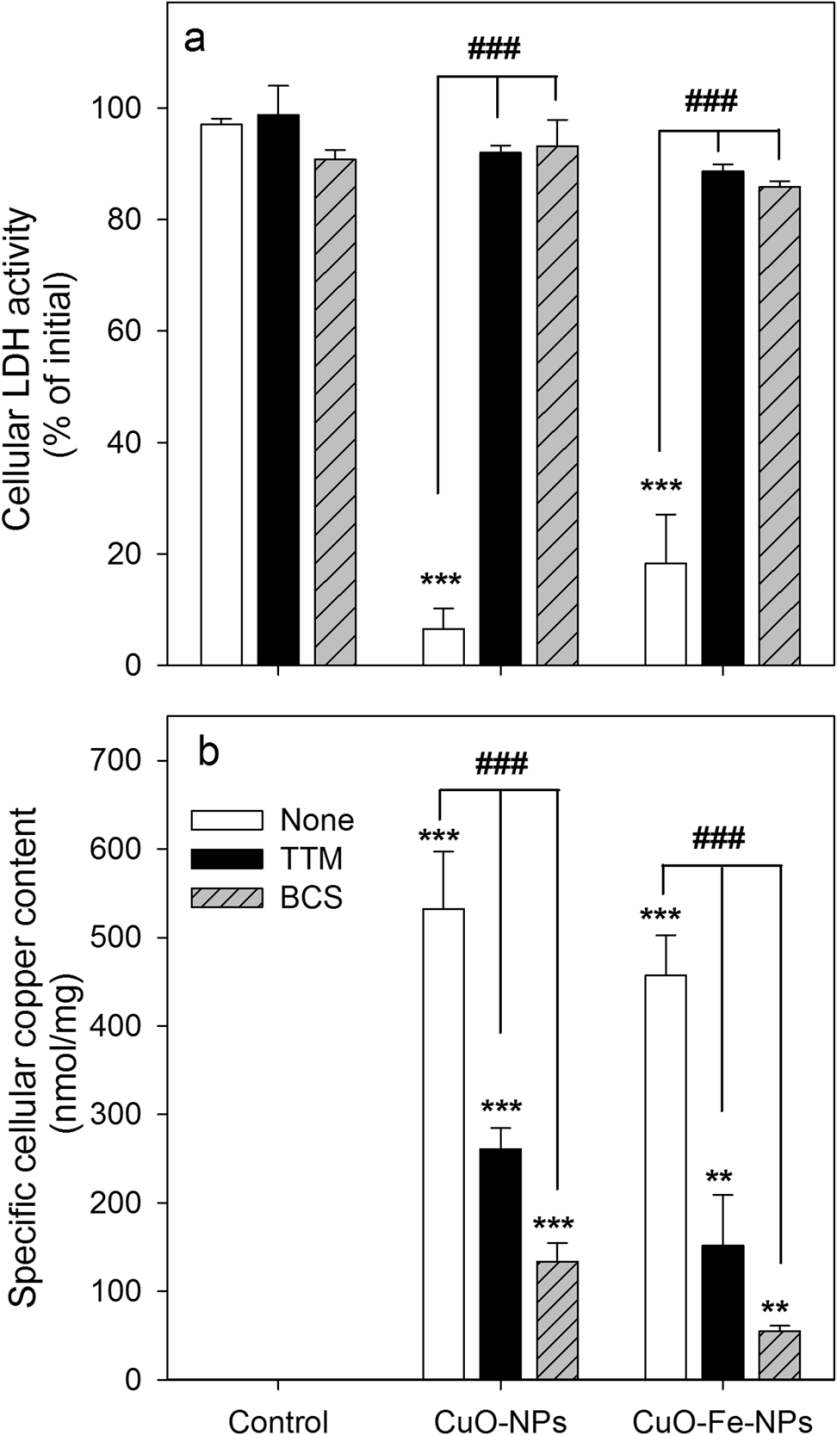
Effects of copper chelators on the viability and the copper content of NP-treated C6 glioma cells. The cells were incubated with 1 mM of CuO-NPs or CuO-Fe-NPs in IB-BSA for 1 h at 37 °C in the absence or the presence of the copper chelators TTM or BCS (2 mM) before the cellular LDH activity (**a**) and the cellular copper content (**b**) were determined. The data shown represent means ± SD of values obtained in three independent experiments. Asterisks indicate the significance of differences of data from cells that had been treated in the absence (control) and in the presence of CuO-NPs or CuO-Fe-NPs (**p < 0.01, ***p < 0.001; ANOVA), whereas hashes indicate the significance of differences of data obtained from cells that had been treated in the absence (none) or the presence of a copper chelator (^###^p < 0.001; ANOVA)

In order to evaluate the effects of copper chelators on the NP-induced toxicity and copper accumulation by C6 cells, cells were incubated with 1 mM CuO-NPs or CuO-Fe-NPs in the absence or the presence of 2 mM of the membrane-permeable (TTM) [56] or membrane-impermeable (BCS) [57] copper chelators for 1 h at 37 °C before the cellular LDH activity and the cellular copper content were determined (Fig. 6). The viability of control cells that had been incubated for 1 h in the absence of NPs without or with copper chelators was not compromised (Fig. 6a) and these cells contained hardly any copper (Fig. 6b). In contrast, cells exposed to CuO-NPs or CuO-Fe-NPs in the absence of chelators were severely damaged (Fig. 6a) and contained high specific copper contents of around 500 nmol/mg (Fig. 6b). Treatment of C6 cells with an excess of the copper chelators TTM or BCS, completely prevented the CuO-NP- and CuO-Fe-NP-induced loss in cellular LDH activity (Fig. 6a) and significantly lowered the cellular copper accumulation of C6 cells for both types of NPs applied by around 50% (TTM) and 80% (BCS) (Fig. 6b).

### Staining for Cellular ROS After Application of CuO-NPs or CuO-Fe-NPs

**Fig. 7:**
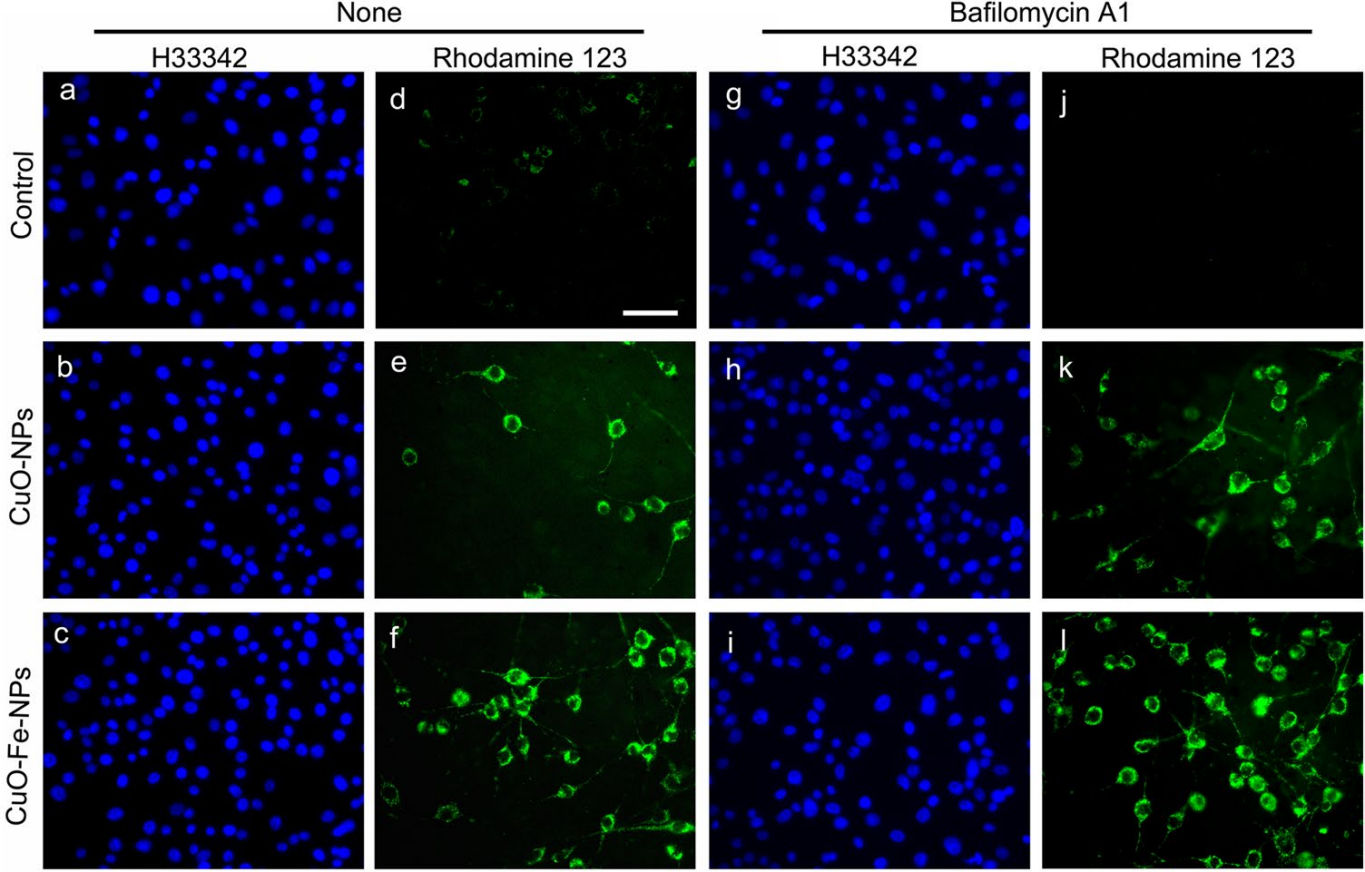
Staining of NP-treated C6 glioma cells for reactive oxygen species (ROS). C6 cells were incubated without NPs (control) (**a**, **d**, **g**, **j**) or with 1 mM CuO-NPs (**b**, **e**, **h**, **k**) or 1 mM CuO-Fe-NPs (**c**, **f**, **i**, **l**) in IB-BSA for 30 min at 37 °C in the absence (none, **a**–**f**) or the presence (**g**–**l**) of 100 nM bafilomycin A1. The cells were subsequently stained for the presence of ROS using the dye dihydrorhodamine 123 (**d**–**f**, **j**–**l**) and the cell nuclei were visualized by staining with the nuclear dye H33342 (**a**–**c**, **g**–**i**). The scale bar in **d** represents 50 µm and applies to all panels

To test whether a treatment with CuO-NP or CuO-Fe-NP leads to an elevated ROS formation in C6 glioma cells, the cells were treated with 1 mM CuO-NPs or CuO-Fe-NPs for 30 min and stained with the dye dihydrorhodamine 123 (Fig. 7). Although these experimental conditions drastically lowered the detectable cellular LDH activity (Figs. 4, 5, 6), almost identical high numbers of cell nuclei were visualized by staining with the membrane permeable dye Hoechst 33342 for control cells and for NP-treated cells (Fig. 7a–c, g–i). While control cells that had not been exposed to CuO-NPs or CuO-Fe-NPs showed only little Rhodamine 123 staining (Fig. 7d), high numbers of ROS-positive cells were found in C6 cell cultures that had been exposed to either CuO-NPs or CuO-Fe-NPs (Fig. 7e, f).

### Bafilomycin A1 Prevents CuO-NP- and CuO-Fe-NP-Induced Toxicity but Not Copper Accumulation and ROS Formation

**Fig. 8:**
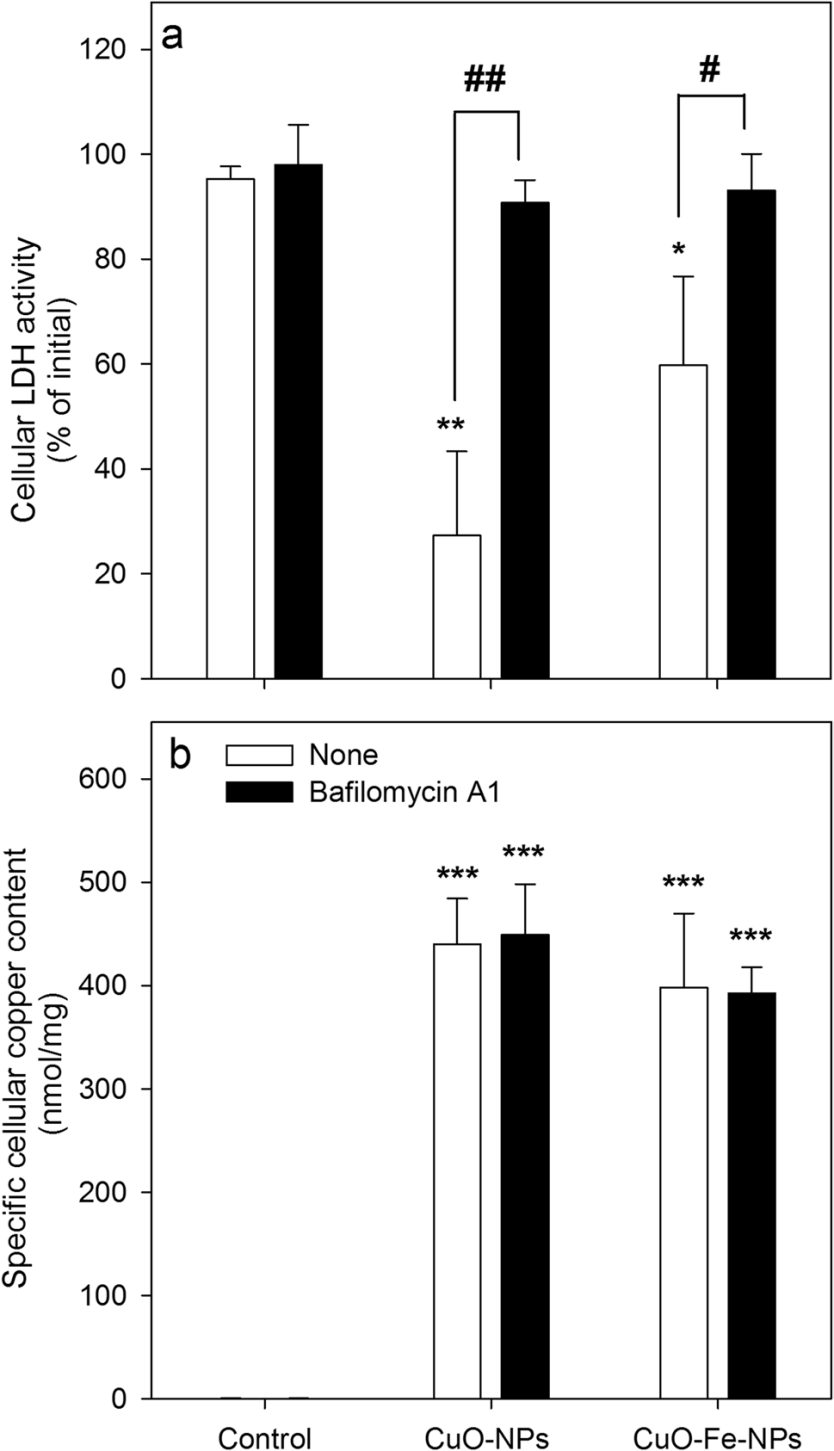
Effects of bafilomycin A1 on the viability and the copper content of NP-treated C6 glioma cells. The cells were incubated without (control) or with 1 mM of CuO-NPs or CuO-Fe-NPs in IB-BSA for 30 min at 37 °C in the absence or the presence of 100 nM bafilomycin A1 before the cellular LDH activity (**a**) and the cellular copper content (**b**) were determined. The data shown represent means ± SD of values obtained in three independent experiments. Asterisks indicate the significance of differences of data obtained for cells treated in the absence (control) and in the presence of NPs (*p < 0.05, **p < 0.01, ***p < 0.001; ANOVA). Hashes indicate the significance of differences of data obtained for cells that had been treated in the absence (none) or presence of bafilomycin A1 (^#^p < 0.05, ^##^p < 0.01; paired *t *test)

In order to evaluate the involvement of lysosomal processes in the adverse effects observed after exposure to CuO-NP- or CuO-Fe-NP, C6 cells were incubated with CuO-NPs or CuO-Fe-NPs in the presence of the lysosomal proton-pump inhibitor bafilomycin A1 which causes the neutralization of the lysosomal pH [58, 59]. The viability of control cells that had been incubated in the absence of NPs was not affected during a 30 min incubation without or with bafilomycin A1 (Fig. 8a) nor did the cells accumulate any copper under such conditions (Fig. 8b). In contrast, the incubation of C6 cells with either CuO-NPs or CuO-Fe-NPs caused a significant loss in cell viability as indicated by the significant decrease in cellular LDH activity to around 20% and 60% of initial values, respectively (Fig. 8a) and to a strong cellular copper accumulation to specific copper contents of around 400 nmol/mg (Fig. 8b). The loss in cell viability after treatment with the NPs was completely prevented in the presence of bafilomycin A1 (Fig. 8a), while the cellular copper accumulation of the exposed cells was not affected by bafilomycin A1 (Fig. 8b). In addition, the presence of bafilomycin A1 did not lower the ROS formation in C6 glioma cells after exposure to CuO-NPs or CuO-Fe-NPs (Fig. 7k, l).

## Discussion

CuO-NPs are known to rapidly release copper ions when dispersed in different types of incubation media [23, 38, 39, 60–62] and the copper ions released have been considered to contribute to the toxicity observed after application of CuO-NPs to cells [24, 28, 29]. As iron-doping of CuO-NPs has been reported to reduce their copper ion release and thereby the CuO-NP-induced cytotoxicity [34], we compared CuO-NPs and CuO-Fe-NPs regarding their dissolution, redox behaviour and toxic potential towards C6 glioma cells. The two types of synthesized NPs were synthesized by flame-spray pyrolysis as previously described [34]. The primary size of the prepared highly crystalline NPs was around 10 nm as analyzed by TEM and was in good agreement with previous TEM and Brunauer–Emmett–Teller analyses performed to measure the surface area of the NPs [34].

The synthesized NPs were coated with DMSA to improve their colloidal stability in physiological media [29, 46] and were dispersed in the protein-containing physiological buffer IB-BSA that was later used for cell experiments. The negative ζ-potential (approximately − 40 mV) of the CuO-NPs and CuO-Fe-NPs dispersed in H_2_O was due to the presence of the cage-like structure of the DMSA coat around the NPs [46, 55]. The ζ-potential of these particles became more positive if dispersed in IB-BSA (around − 12 mV) which is most likely the consequence of the formation of a protein corona around the particles, which has been discussed to further enhanced the colloidal stability of the NPs in physiological medium [63–65]. The mean hydrodynamic diameter of the dispersed particles in H_2_O or in IB-BSA (approximately 200–250 nm) was in accordance with literature data for dispersions of these particles [34]. Notably, the coated CuO-NPs and CuO-Fe-NPs synthesized and applied to the cells did not differ in size or ζ-potential, demonstrating that the doping of CuO-NPs with 10% iron did not alter the physicochemical properties of these NPs.

Quantification by AAS of the iron content of the synthesized NPs confirmed that CuO-NPs contained only negligible amounts of iron while analysis of CuO-Fe-NPs revealed that these NPs contained iron in amounts that accounted to 10% of the determined copper content. Also the analysis of extracts of cells that had been exposed to CuO-Fe-NPs contained around 10% iron compared to the copper determined for the respective lysates, demonstrating that intact CuO-Fe-NPs had been taken up into the cells.

The reported higher stability of CuO-Fe-NPs compared to iron-free CuO-NPs was tested for dispersions of the NPs in IB-BSA. The more rapid initial formation of a copper-BCS complex after application of BCS to dispersions of CuO-NPs compared with CuO-Fe-NPs is consistent with a higher lability of CuO-NPs to release copper ions. However, during longer incubations an excess of BCS liberated copper almost completely from both types of NPs as also reported earlier for other types of DMSA-coated CuO-NPs [29, 30], demonstrating that the presence of iron ions in the crystalline structure of CuO-Fe-NPs does only gradually slow down, but not prevent, the release of copper ions at least in the presence of BCS.

Analysis of the redox activity of CuO-NPs and CuO-Fe-NPs by cyclic voltammetry measurements in IB-BSA revealed a similar redox-behavior on the NP surface as reported for these CuO-NPs dispersed in other media [34] and confirmed differences between the two types of NPs analyzed. The pronounced oxidation peak (A1) observed for CuO-NPs is characteristic for interaction of CuO-NPs with amino acids (data not shown), suggesting a redox reaction between the CuO-NP surface and BSA present in IB-BSA. After the addition of BCS, the interaction of CuO-NPs with IB was substantially reduced most likely due to the chelation of liberated copper ions from the medium. For the CuO-Fe-NP electrodes, similar redox-reactions were observed as for CuO-NP electrodes, but lower reaction intensities were recorded. This substantial reduction in the interaction between CuO-Fe-NPs and the physiological medium applied, compared to the respective CuO-NPs setting, is consistent with a lower release rate of copper ions from the iron-doped NPs due to the presence of iron in the crystal structure of the CuO-Fe-NPs [34].

To compare CuO-NPs and CuO-Fe-NPs concerning the copper accumulation and toxic potential in C6 glioma cells, identical copper concentrations were applied as NP dispersions under identical experimental conditions. Both types of NPs caused a time-, concentration- and temperature-dependent copper accumulation and impairment of cell viability, as demonstrated previously for C6 glioma cells that had been exposed to other types of DMSA-coated CuO-NPs [29, 30]. Copper accumulation was strongly lowered and toxicity was prevented in the presence of an excess of copper chelators as well as by lowering the incubation temperature which is also consistent with literature data [29, 30]. Likely reasons for these observations are the disintegration of the NPs in presence of copper chelators (present report, [29]; and the inhibition of endocytotic uptake of NPs at a temperature of 4 °C [30, 46, 55].

For all experimental conditions applied in this study, almost identical specific copper contents were determined for C6 cells that had been exposed to CuO-NPs or CuO-Fe-NPs under identical conditions. Thus, binding of the NPs to the cells as well as internalization of the bound NPs appears to be identical for both types of NPs investigated, confirming that due to the almost identical size and surface charge the cells were unable to discriminate between the two types of NPs applied for this study. However, despite identical specific copper contents determined for cells that had been exposed to either CuO-NPs or CuO-Fe-NPs, the exposure of C6 cells to iron-free NPs compromised the cell viability more strongly compared to the respective CuO-Fe-NP condition, as also previously reported for other biological test systems [34]. This is likely to be a direct consequence of the higher stability of iron-doped CuO-NPs which results in a reduced release of copper ions [34].

Several studies demonstrated that the cytotoxic potential of CuO-NPs is a result of the intracellular release of copper ions from internalized CuO-NPs which takes place in the strongly reducing lysosomal environment [20–22, 66] and which subsequently lead to severe oxidative stress [13, 14]. Also for the toxicity observed for CuO-NPs and CuO-Fe-NPs on C6 glioma cells, the low pH of the lysosomal compartment appears to be crucial as neutralization of the lysosomal pH by application of the lysosomal proton-pump inhibitor bafilomycin A1 [59, 67], completely prevented the NP-induced toxicity in C6 cells while this inhibitor did not affect cellular copper accumulation. Thus, not the cellular uptake of NPs but rather the mobilization of copper from the internalized NPs in the acidic environment of the lysosomes appears to be the crucial step responsible for the toxicity of copper-containing NPs in C6 glioma cells.

Despite of the prevention of the toxicity induced by CuO-NPs and CuO-Fe-NPs by bafilomycin A1, the formation of cellular ROS in the treated cells was not affected, suggesting that not the ROS-induced oxidative stress but rather other adverse consequences of an exposure to copper-containing NPs are mainly responsible for the observed toxicity. A similar persistence of cellular ROS formation in CuO-NPs-treated C6 glioma cells under conditions which prevented toxicity has recently been reported [30]. Thus, ROS formation and oxidative stress appears to contribute little to the toxicity observed for CuO-NP-treated C6 cells. More likely other adverse processes which have been linked to the cellular presence of CuO-NPs or copper ions liberated from internalized NPs are responsible for the toxicity observed after application of CuO-NPs or CuO-Fe-NPs to C6 cells. Such processes may include the inactivation of cellular enzymes, disturbances of protein folding, protein aggregation, thiol cross-linking as well as impairment of lysosomal functions [68–71]. The more severe toxicity observed after a treatment of C6 glioma cells with iron-free CuO-NPs compared to CuO-Fe-NPs is likely to be caused by the more rapid release of copper ions from internalized iron-free CuO-NPs, which is associated by a more severe inactivation of crucial cellular processes.

In conclusion, DMSA-coated CuO-NPs and CuO-Fe-NPs did not differ in their physicochemical properties and were accumulated by C6 glioma cells with almost identical rates. Both types of NPs compromised the viability of C6 cells, but the CuO-Fe-NPs had a lower toxic potential than iron-free CuO-NPs which is consistent with lower release of copper ions and a reduced surface redox chemistry of the iron-doped NPs. The growing production of CuO-NPs for a variety of applications [72–75] has increased the risk of environment damage and exposure of humans to CuO-NPs, which has raised major concerns for their adverse impacts [76–79]. In this context, iron-doped CuO-NPs might represent a less toxic alternative to the more rapidly dissolving CuO-NPs, which are known for their severe adverse effects in a number of biological systems [80, 81]. Furthermore, a controlled copper ion release from CuO-NPs by iron-doping can have interesting therapeutic potential as very recently demonstrated for an application of iron-doped CuO-NPs in anti-cancer treatment [82].
